# Integrated effects of vermicompost and town refuse on growth and nutritional status of onion cultivated in calcareous soil

**DOI:** 10.1038/s41598-026-38173-8

**Published:** 2026-03-07

**Authors:** Elham A. Badr, Saied El Sayed, Magda H. Mohamed

**Affiliations:** 1 Field Crops Department, National Research Centre, 33 El-Behouth St, 12622, Dokki, Cairo, Egypt; 2 Plant Nutrition Department, National Research Centre, 33 El-Behouth St, 12622, Dokki, Cairo, Egypt

**Keywords:** Nutrient content-Onion plant, Town refuse -Vermicompost, Environmental sciences, Physiology, Plant sciences

## Abstract

This study investigates the influence of organic amendments vermicompost and town refuse on the growth parameters, nutrient uptake, and biochemical quality of onion (*Allium cepa L)* grown in calcareous soils. A pot experiment was carried out at Burg El-Arab City, Alex, Egypt. By using three levels (10, 15, and 20 ton fed^-1^) of both amendments compared to a non-treated control. Results indicated that the application of vermicompost, particularly at 20 ton fed^-1^ significantly enhanced chlorophyll a, b and carotene levels (0.086, 0.036 and 0.131mg g^-1^ FW) as well as growth indicators such as plant height, bulb diameter, and total leaf area (44.58 cm, 4.268 cm and 2.894 cm^2^. Both fresh and dry weights of bulbs and leaves increased notably with organic treatments. Macronutrient (N, P, K) and micronutrient (Fe, Zn, Cu) concentrations and uptake were significantly elevated by increasing amendment levels, with vermicompost showing superior results over town refuse. Additionally, the oil content (9.83%), total soluble sugars (TSS) (15.25%), and protein percentage (28.95%) in onion (*Allium cepa L*) bulbs improved remarkably under organic treatments. Strong positive correlations were found among most growth and nutrient parameters, indicating that enhanced nutrient availability translates directly to improve physiological and yield responses. The findings suggest that vermicompost is an effective and sustainable amendment for improving onion (*Allium cepa L*) productivity and nutritional quality under nutrient-poor calcareous soil conditions.

## Introduction

Onion (*Allium cepa L.*) is one of the most widely cultivated vegetable crops, valued for its nutritional, culinary, and medicinal properties. Enhancing its productivity is vital to meet the growing food demand and improve farmers’ income. However, sustaining onion production is often constrained by declining soil fertility and overreliance on chemical fertilizers.

- Vermicompost offers an environmentally and economically friendly solution for soil amendment. It is the decomposition of organic matter by oligochaete worms—such as *Lampito mauritii*, *Eudrilus eugeniae*, *Perionyx excavatus*, and *Eisenia foetida*—which convert materials like manure, coffee husks, and plant residues into nutrient-rich compost via their digestive processes^1,2^. The resulting vermicompost not only enriches the soil with bioavailable nutrients but also improves its structure, moisture retention, and microbial activity, making it a valuable biofertilizer.
- In contrast, the use of town refuse decomposed organic waste from urban households and markets presents an additional avenue to recycle biodegradable waste into agriculture. When adequately treated, town refuse can provide a cost-effective source of organic matter and nutrients to improve soil fertility. While inorganic fertilizers are critical for increasing crop yields and ensuring food security^3^ their excessive and prolonged use can degrade soil quality, harm beneficial soil organisms, and pose risks to environmental and human health^4^. Therefore, integrating organic inputs like vermicompost and town refuse with or as alternatives to chemical fertilizers have gained attention as a sustainable strategy. Research has shown that sole application of either organic or inorganic fertilizers may not sufficiently maintain soil productivity; hence, combining both is essential to sustain soil health and optimize nutrient use efficiency^5^.
- Organic fertilizers, including vermicompost, can act as crucial sources of nutrients for plant growth. Furthermore, organic fertilizers can improve soil quality by maintaining soil structure, supplying more beneficial microorganisms, increasing soil water holding capacity, increasing microfauna’ density, improving soil pH, and improving the stability of earthworm communities^6^. Consequently, agricultural practices with organic fertilization can improve the growth; productivity and quality of crops^7^.
- Vermicompost has also shown their potential for suppressing diseases and pests in agricultural crops. Such suppression may be related to many mechanisms, such as a higher density of useful microbes or total microbiological activity, which prohibits pathogens or pests, induces systemic resistance in plants against pests/diseases, improves soil quality and provides essential nutrients for pest control, and increases the competition of useful microbes for food/space/water with harmful microbes^8^.

Given this background, the present study aims to evaluate the effects of vermicompost and town refuse on the growth and yield performance of onion (*Allium cepa* L). This research will contribute to identifying sustainable soil fertility management practices that enhance crop productivity while minimizing environmental risks.

## Materials and methods

A pot experiment was carried out at Burg El-Arab City, Alex, Egypt to study the effect of Vermicompost and Town refuse the growth parameter and chemical composition of onion (*Allium cepa* L.). To evaluate the response of onion plants to different types of organic fertilizers. Soil sample was taken before transplanting, air-dried, sieved by 2 mm sieve and analyzed. Some chemical and physical properties are present in Table 1. The experiment contained 21 plastic pots having a diameter of 20 cm and length of 25 cm. each pot filed with 8 kg soil. Seedlings of onion plants (*Allium cepa* L) were obtained from Vegetable Department, Ministry of Agriculture. Uniform onion seedlings cv. Giza 20 at 4 to 5 green true leaf stage was transplanted at November 2022. The full amounts of phosphorus were applied at the time of final preparation. However, nitrogen fertilizer at 80 kg N fed^− 1^ as ammonium nitrate (33.5%) was side dressed in two equal portions at 30 and 60 days after transplanting date. Potassium fertilizer at 50 kg K_2_O fed^− 1^ was applied as potassium sulphate (46%) at in two equal portions at 35 and 45 days after transplanting date. Organic matter using three rates 10, 15 and 20 ton fed^− 1^ from two sources (Vermicompost and Town refuse), some chemical and physical properties are present in Table 2.With three replicates for each treatment and each pot with three transplants of onion.

**Table 1:** Some characteristics of the investigated soil.

Physical properties (%)			Texture	Chemical properties						
				pH (1:1)	EC (1:1) dSm^− 1^	CaCO_3_ %	OM %	Available nutrient (mg/kg)		
Sand	Silt	Clay	Sandy clay loamy					**N**	**P**	**K**
57.82	21.59	20.59		8.31	2.56	28.9	0.52	16.7	5.9	45.92

**Table 2:** Chemical composition of some macro- and micronutrients of vermicompost and town refuse fertilizers used in the experiment study.

Analysis			Vermicompost	Town refuse
pH	(1:10)		8.15	6.95
EC	dS/m		5.24	7.88
OM	%		29.85	28.90
C/N	ratio		12.14	22.80
N	%		1.43	1.48
P	%		0.94	0.46
K	%		0.52	0.83
Fe	ppm		775	1346
Mn	ppm		132	239
Zn	ppm		41.6	250
Cu	ppm		12.7	93
***The experimental treatments were as follows***:				
**T1**: Control				
**T2**: Vermicompost 10 (ton fed^− 1^)		**T5**: Town refuse 10 (ton fed^− 1^)		
**T3**: Vermicompost 15 (ton fed^− 1^)		**T6**: Town refuse 15 (ton fed^− 1^)		
**T4**: Vermicompost 20 (ton fed^− 1^)		**T7**: Town refuse 20 (ton fed^− 1^)		

### Recorded data

After 120 days at maturity stage, the following data were taken: Plant height, fresh and dry matter of leaves Root length (m), bulb diameter (cm) and total leave area (cm^2^**)**.

### Chemical constituents

- Chlorophyll a, b and total carotenoids in leaves after 80 days from sowing were determined using the method described by^9^.
- Extraction of essential oil: bulb Hydro distillation for 3 h using a Clevenger type apparatus^10^.
- Total Nitrogen, Phosphorus, Potassium, Sodium and Calcium in fresh and dry matter of leaves and bulb were determined according to the methods of the^11^. Iron, zinc and copper contents were determined using atomic absorption spectrophotometer^11^.
- Total soluble sugars (TSS) extracted according to^12^ and assayed according to^13^.
- The physical and chemical properties of the soil were determined according to the method described by^14^.
- Crude protein percentage was extracted and determined by Micro-Kjeldahl method as described by^15^. The value of total crude protein was calculated by multiplying total values of total-N by factor 6.25.

### Statistical analysis

The means of data recorded were subjected to the analysis of variance according to^16^. The Least Significant Differences (LSD) at *P* = 0.05 level was used to verify the differences among means of the treatments.

## Results

### Photosynthetic pigments

Chlorophyll a content increased from 0.086 mg g^−1^ FW in the control to 0.110 mg g^−1^ F.W with 20 ton fed^−1^ vermicompost, and 0.103 mg g^−1^ FW with 20 ton fed^−1^ town refuse. Chlorophyll b followed a similar trend, increasing from 0.036 mg g^−1^ F.W (control) to 0.062 mg g^−1^ FW (vermicompost 20 ton fed^−1^). Total chlorophyll (a + b) content peaked at 0.172 mg g F.W with vermicompost (20 ton fed^−1^), with significant increases across all treatments. Carotene content ranged from 0.076 (control) to 0.131 mg g F.W with vermicompost (20 ton fed^−1^), indicating enhanced pigment biosynthesis.

### Growth parameters

Table 3 indicated that the application of vermicompost and town refuse significantly enhanced all measured growth parameters of onion plants grown in calcareous soil when compared to the control. The improvement was generally proportional to the rate of application (10, 15, and 20 ton fed^1^, with the 20 ton fed^−1^ of vermicompost consistently recording the highest values across most parameters.

#### Growth and biomass

Plant height was significantly greater in amended treatments, with the tallest plants (44.58 cm) in vermicompost (20 ton fed^−1^) and the shortest (31.52 cm) in the control. Fresh weight of bulb improved from 14.10 g (control) to 19.26 g with application of vermicompost (20 ton fed^−1^), and 18.40 g with applied town refuse at a rate of (20 ton fed^−1^). Fresh weight of leaves was highest with vermicompost (20 ton fed^−1^) at 40.86 g, compared to 21.76 g in the control. Dry bulb and leaf weights were also enhanced, peaking at 4.010 g and 9.226 g, respectively, with application of vermicompost (20 ton fed^−1^).

#### Root development

Root length significantly improved with organic amendments. The longest roots (58.06 cm) were observed in vermicompost (20 ton fed^−1^), while the control showed the shortest roots (32.73 cm). All differences among treatments were statistically significant at *P* ≤ 0.05, as indicated by LSD values for each parameter. Plant height, biomass, and root development were strongly influenced by the application of vermicompost and town refuse, with vermicompost showing the strongest effect across all measured parameters.

**Table 3:** Photosynthetic pigments and growth parameters of onion plant as affected by vermicompost and town refuse grown in calcareous soil.

Characterization	Control	Vermicompost (ton fed^− 1^)			Town refuse (ton fed^− 1^)			LSD 0.05
		10	15	20	10	15	20	
*Chlorophyll a (mg/g f.w)*	0.086	0.097	0.099	0.110	0.092	0.096	0.103	*0.0066*
*Chlorophyll b (mg/g f.w)*	0.036	0.045	0.052	0.062	0.043	0.045	0.055	*0.0053*
*Chlorophyll (a + b) (mg/g f.w)*	0.122	0.142	0.151	0.172	0.135	0.141	0.158	*0.0044*
*Carotene (mg/g f.w)*	0.076	0.096	0.108	0.131	0.083	0.094	0.117	*0.0094*
*Plant height (cm)*	31.52	39.41	42.36	44.58	35.12	37.14	39.55	*0.5327*
*Fresh weight of Bulb (g)*	14.10	17.10	18.43	19.26	15.66	16.26	18.40	*0.3460*
*Fresh weight of Leaves (g)*	21.76	32.60	33.66	40.86	29.76	31.26	38.53	*1.4226*
*Dry weight of Bulb (g)*	2.237	3.068	3.481	4.010	2.403	2.946	3.671	*0.1506*
*Dry weight of Leaves (g)*	6.272	7.117	7.596	9.226	6.285	6.833	8.711	*0.3488*
*Root Length (m)*	32.73	40.49	47.77	58.06	34.82	36.56	49.36	*2.0174*

#### Bulb diameter and total leave area of onion plant

**Fig. 1:**
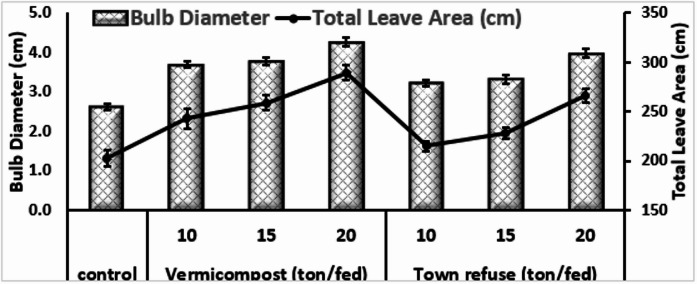
Bulb diameter and Total Leave Area of Onion plant as affected by Vermicompost and Town refuse grown in calcareous soil.

Application of vermicompost and town refuse resulted in a significant improvement in bulb diameter and total leaf area compared with the control (Fig. 1). The control treatment exhibited the lowest bulb diameter 2.620 cm and totals leaf area 202.750 cm². The greatest enhancement was recorded under vermicompost application at 20 ton fed⁻¹, which produced the largest bulb diameter 4.268 cm and the maximum total leaf area 289.4 cm². Similarly, town refuse applied at 20 ton fed⁻¹ significantly improved both traits, reaching 3.980 cm for bulb diameter and 266.4 cm² for total leaf area, although the magnitude of increase was still lower than that recorded with vermicompost. A clear and consistent upward trend was observed with increasing application rates of both organic amendments.

### Chemical constituents

#### Macronutrient contents and uptake of onion plant

Table 4 illustrates the effect of different rates of vermicompost and town refuse on the macronutrient content (N, P, K) and uptake by onion plants (bulb and leaves) in calcareous soil. Nitrogen content in both bulb and leaves significantly increased with the application of vermicompost, reaching maximum values at 20 ton fed^−1^ (2.96% in bulbs and 1.99% in leaves) compared to the control (1.87% and 0.98%, respectively). Phosphorus content followed a similar trend, peaking at (0.52% in bulbs and 0.41%) in leaves under 20 ton fed^−1^ vermicompost. Potassium content was highest in bulbs (2.88%) and leaves (4.15%) at 20 ton fed^−1^. Vermicompost, compared to 1.80% and 3.17% in the control, respectively. Nutrient uptake (mg kg^−1^) also increased markedly with the application of organic amendments. The highest N uptake in bulbs and leaves was 118.7 and 183.3 mg kg^−1^ respectively under vermicompost 20 ton fed^−1^. Similarly, P uptake peaked at 20.93 mg kg^−1^ in bulbs and 37.71 mg kg^−1^ in leaves, while K uptake reached 115.54 and 383.1 in leaves mg kg^−1^ under the same treatment.

**Table 4:** Macronutrient content and uptake of onion plant as affected by vermicompost and town refuse grown in calcareous soil.

Characterization	Control	Vermicompost (ton fed^− 1^)			Town refuse (ton fed-1)			LSD 0.05
		10	15	20	10	15	20	
*Total N of Bulb (%)*	1.87	2.08	2.63	2.96	1.83	2.16	2.89	***0.1562***
*Total N of leaves (%)*	0.98	1.59	1.69	1.99	1.15	1.41	1.88	***0.0832***
*Total P of Bulb (%)*	0.24	0.41	0.46	0.52	0.28	0.33	0.50	***0.0187***
*Total P of leaves (%)*	0.20	0.28	0.34	0.41	0.23	0.25	0.37	***0.0191***
*Total K of Bulb (%)*	1.80	2.37	2.61	2.88	2.00	2.17	2.68	***0.0138***
*Total K of leaves (%)*	3.17	3.71	3.83	4.15	3.17	3.39	4.14	***0.0413***
*N uptake of Bulb (mg/kg)*	41.7	63.9	91.4	118.7	44.0	63.6	106.1	***0.2360***
*N uptake of leaves (mg/kg)*	61.7	113.3	128.2	183.3	72.4	96.6	163.9	***1.2640***
*P uptake of Bulb (mg/kg)*	5.38	12.50	15.93	20.93	6.75	9.81	18.22	***0.0821***
*P uptake of leaves (mg/kg)*	12.67	20.25	25.80	37.71	14.57	17.14	32.42	***0.3120***
*K uptake of Bulb (mg/kg)*	40.21	72.78	90.89	115.54	48.09	63.90	98.28	***0.4570***
*K uptake of leaves (mg/kg)*	198.8	264.3	291.0	383.1	199.1	231.6	360.3	***2.0924***

#### Micronutrient contents and uptake of onion plant

Table 5 shows that application of vermicompost and town refuse significantly improved the micronutrient content (Fe, Zn, and Cu) and uptake in onion bulbs and leaves compared to the control. Iron (Fe). Bulb content increased from 332 ppm (control) to 579 ppm with 20 ton fed^− 1^ vermicompost. Leaf content increased from (640) ppm to 881 ppm under the same treatment. Fe uptake peaked at 2322 mg kg^− 1^ in bulbs and 8131 mg kg^− 1^ in leaves under 20 ton fed^− 1^ vermicompost. Zinc (Zn).Bulb content increased from 126 ppm (control) to 195 ppm. Leaf content increased from 76.3 ppm to 106.8 ppm. Maximum Zn uptake by bulbs and leaves was 780 mg kg^− 1^ and 985 mg kg^− 1^, respectively, at 20 ton fed^− 1^ vermicompost. Copper (Cu). Bulb content increased from 15.4 ppm to 22.1 ppm. Leaf content increased from 9.8 ppm to 16.8 ppm. Cu uptake reached 88.8 mg kg^− 1^ in bulbs and 155.1 mg kg^− 1^ in leaves under 20 ton fed^− 1^ vermicompost.

Town refuse also enhanced micronutrient content and uptake, though to a slightly lesser extent than vermicompost. The improvements were statistically significant compared to the control for all treatments, with the LSD (0.05) values confirming meaningful differences. Vermicompost at 200 ton fed^− 1^ consistently providing the best results.

**Table 5:** Micronutrient content and uptake of onion plant as affected by vermicompost and town refuse grown in calcareous soil.

Characterization	Control	Vermicompost (ton fed^− 1^)			Town refuse (ton fed^− 1^)			LSD 0.05
		10	15	20	10	15	20	
*Total Fe of Bulb (ppm)*	332	441	541	579	406	426	565	*22.42*
*Total Fe of leaves (ppm)*	640	817	844	881	719	764	863	*38.21*
*Total Zn of Bulb (ppm)*	126	141	153	195	132	139	192	*7.079*
*Total Zn of leaves (ppm)*	76.3	90.8	98.0	106.8	80.7	86.8	101.7	*4.408*
*Total Cu of Bulb (ppm)*	15.4	18.1	19.1	22.1	16.2	17.4	21.6	*0.889*
*Total Cu of leaves (ppm)*	9.8	12.4	12.7	16.8	10.1	10.8	14.0	*0.805*
*Fe uptake of Bulb (mg/kg)*	743	1352	1883	2322	975	1254	2074	*26.31*
*Fe uptake of leaves (mg/kg)*	4012	5816	6409	8131	4516	5221	7521	*32.61*
*Zn uptake of Bulb (mg/kg)*	282	433	531	780	317	409	703	*4.135*
*Zn uptake of leaves (mg/kg)*	478	646	744	985	507	593	886	*2.698*
*Cu uptake of Bulb (mg/kg)*	34.4	55.5	66.5	88.8	38.9	51.1	79.3	*0.716*
*Cu uptake of leaves (mg/kg)*	61.2	88.1	96.6	155.1	63.5	74.1	121.9	*0.659*

#### Oil content, total soluble sugar and protein content of onion plant

**Fig. 2:**
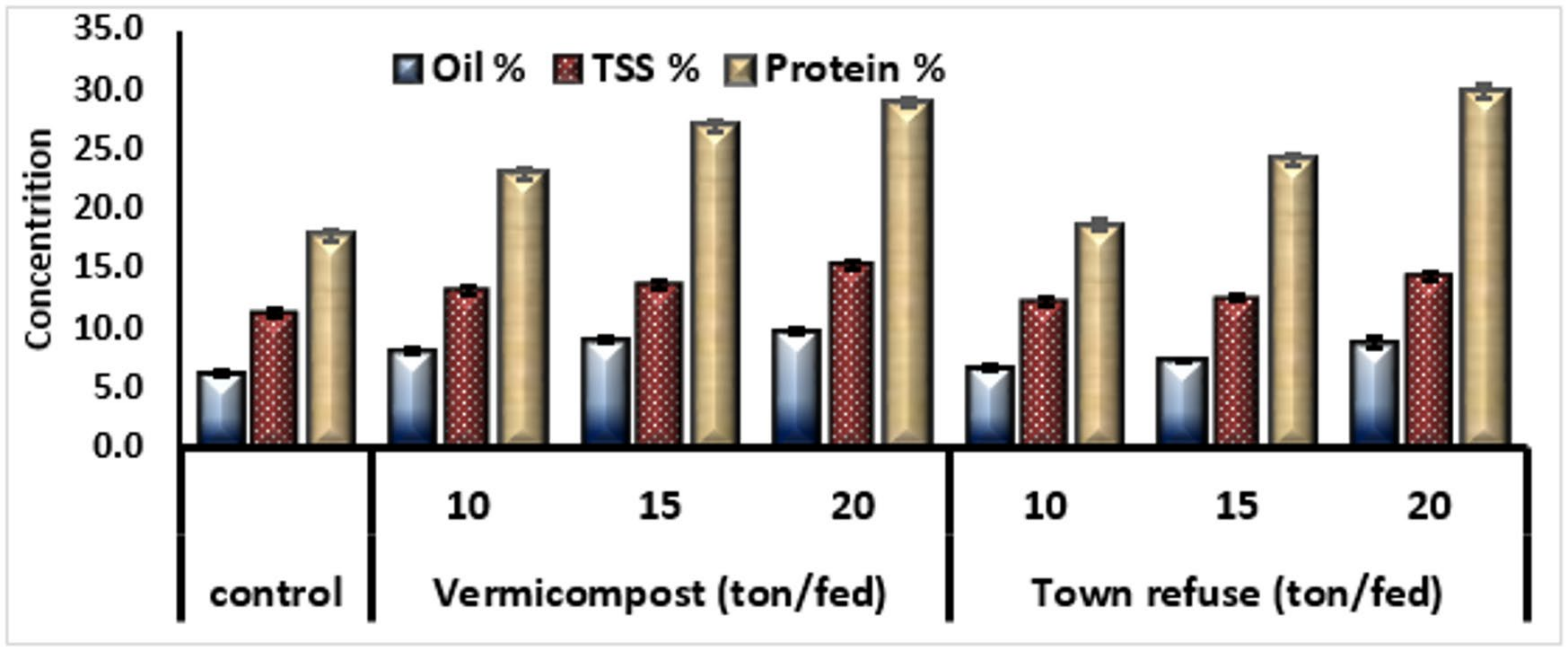
Oil content, total soluble sugar and protein content of Onion plant as affected by Vermicompost and Town refuse grow in calcareous soil.

Figure (2). The data clearly demonstrate that both vermicompost and town refuse are effective organic amendments for improving the biochemical quality of onion bulbs in calcareous soils. The application of Vermicompost and Town Refuse significantly enhanced the oil content, total soluble sugar (TSS) and protein content of onion bulbs compared to the control. Oil content increased from 6.237% (control) to a maximum of 9.830% with 20 ton fed^−1^ Vermicompost, and 8.850% with 20 ton fed^−1^ Town refuse. oil Content. While it became clear that TSS (%) rose from 11.285% in the control to 15.253% at 20 ton fed^−1^ Vermicompost and 14.370% with 20 ton fed^−1^ Town refuse. Total Soluble Sugar, an important quality parameter in onions, improved significantly with organic treatments. Vermicompost, especially at 20 ton fed^−1^, showed the highest TSS. Protein content showed a remarkable increase from 17.80% (control) to 28.95% and 29.83% at 20 ton fed^−1^ Vermicompost and Town refuse, respectively.

### Correlation

Data in Table 6 show that correlation coefficients between growth parameters and nutrient content of onion plants as affected by vermicompost and town refuse in calcareous soil. The correlation matrix (Table 6) reveals strong and positive relationships between growth parameters (fresh weight, dry weight, bulb diameter, total leaf area, and root length) and nutrient content (N, P, K, Fe, Zn, Cu), as well as oil and TSS percentages. Strongest correlations were observed between. Fresh Weight (F.W B) and Potassium (K% %) (*r* = 0.992). Total Leaf Area (TLA**)** and Dry Weight (D.W B) (*r* = 0.990). Oil% **%** and Total Leaf Area (*r* = 0.990). TSS **%** and Bulb Diameter (*r* = 0.982). All correlations between oil content and nutrient/growth parameters were strong and positive, with values ranging from 0.928 to 0.994. Similarly, TSS**%** showed significant positive correlations with all studied traits, particularly with Potassium (*r* = 0.984) and TLA (*r* = 0.991). The high and positive correlation coefficients across most parameters suggest a synergistic effect of organic amendments on onion plant growth, nutrient uptake, and biochemical quality.

**Table 6:** Correlation effect between of growth parameters and nutrient content of onion plant as affected by vermicompost and town refuse grown in calcareous soil.

D.W (B) g	F.W (B) g	D.W (B) g	B Diam	TLA (cm)	Root L (m)	*N* %	*P* %	K %	Fe ppm	Zn ppm	Cu ppm	Oil %
	0.975											
B Diam	0.981	0.954										
TLA (cm)	0.978	0.990	0.967									
Root L (m)	0.944	0.965	0.923	0.986								
N %	0.915	0.962	0.870	0.947	0.954							
P %	0.982	0.985	0.967	0.984	0.953	0.942						
K %	0.992	0.991	0.975	0.994	0.970	0.943	0.994					
Fe ppm	0.981	0.967	0.949	0.965	0.949	0.958	0.970	0.978				
Zn ppm	0.857	0.912	0.866	0.917	0.928	0.951	0.899	0.897	0.907			
Cu ppm	0.938	0.973	0.939	0.973	0.961	0.969	0.968	0.966	0.957	0.978		
Oil %	0.986	0.984	0.958	0.990	0.969	0.928	0.985	0.994	0.961	0.860	0.940	
TSS %	0.971	0.978	0.982	0.991	0.971	0.930	0.973	0.984	0.961	0.935	0.980	0.967

### Discussion

The present study demonstrated that the application of vermicompost and town refuse markedly improved the physiological, growth, and biochemical characteristics of onion plants cultivated in calcareous soils (Table 3). These soils are typically characterized by poor organic matter content, high pH, and low nutrient availability; therefore, the substantial improvement observed across all parameters signifies the effectiveness of organic amendments in overcoming such constraints. The significant increases in chlorophyll a, chlorophyll b, total chlorophyll, and carotene under vermicompost and town refuse.Treatments reflect enhanced chloroplast development and improved nutrient supply particularly nitrogen and magnesium, which are essential components of the chlorophyll molecule. Vermicompost exerted a superior effect, likely due to its high microbial activity, humic substances, and balanced nutrient composition that enhance nutrient mineralization and uptake. These findings agree with earlier reports that vermicomposting stimulates pigment biosynthesis and photosynthetic efficiency in various crops^17^. illustrated that pepper grown in potting mixtures containing 40%food waste vermicompost and 60% MM360 yielded 45% more fruit weights and had 17% greater mean number of fruits than those grown in MM 360 only. Moreover, the earlier study of^18,19^. Showed that vermicompost at rate (25 ml L^−1^) increased the contents of chlorophyll a, b and activity of peroxidase and polyphenol oxidase in onion plant leaves. Additionally the substantial increases in plant height, biomass, and root length particularly under the 20 ton fed⁻¹ vermicompost treatment, indicate improved physiological performance and better soil–plant interactions. Vermicompost is known to enhance soil structure, water-holding capacity, and microbial diversity, which collectively contribute to greater nutrient availability and enhanced root proliferation. These results align with findings by^20,21^, who reported significant improvement in plant morphology with vermicompost application. Although town refuse also improve the growth parameters, its effect was slightly lower, likely due to its slower decomposition rate and less uniform nutrient profile. The superior performance of vermicompost can be attributed to its higher biological activity and faster nutrient mineralization rate, which support enhanced shoot and root biomass production compared with town refuse. Vermicompost also improves soil microbial diversity and enzymatic activities, contributing to better nutrient cycling, increased root penetration, and more efficient water uptake^20^. Previous studies support these findings^21^. reported that a 50% vermicompost + 50% soil mixture significantly increased shoot length, internodal length, and the number of leaves and branches in *Capsicum annuum*. Similarly^22^, found that combining vermicompost with peat improved plant height and leaf number in cucumber plants. In the present study, plant growth consistently increased with higher vermicompost application rates. Although town refuse also produced positive effects, its impact was comparatively lower, likely due to its slower decomposition, reduced microbial activity, and less balanced nutrient composition. Nevertheless, at 20 ton fed⁻¹, it performed significantly better than the control, indicating that it remains a practical, low-cost soil amendment for farmers with limited resources. The observed increase in dry matter accumulation and root length reflects a more extensive and efficient root system, capable of enhancing nutrient and water uptake—an essential factor for improving overall plant performance. Different treatments caused significant improvements in bulb diameter and leaf area (Fig. 1) resulting from vermicompost and town refuse applications can be attributed to enhanced nutrient availability, improved soil structure, and better root development. Calcareous soils are often characterized by high pH and poor nutrient availability; however, organic amendments play a major role in improving soil aeration, water-holding capacity, and microbial activity, thereby supporting better plant growth. Vermicompost is particularly effective due to its rich content of readily available macronutrients (N, P, K) and biologically active compounds such as auxins, cytokinins, and gibberellins. These substances stimulate cell division and expansion, which directly enhances bulb formation and leaf tissue development. Although town refuse compost also improves growth parameters, its heterogeneous organic composition may lead to slightly reduced efficiency compared with vermicompost. The increase in total leaf area is of particular importance, as it contributes to greater photosynthetic capacity and subsequently supports enhanced bulb enlargement and overall plant vigor. These findings agree with previous studies. reported significant improvements in vegetative growth and yield attributes of various crops with vermicompost applications. Similarly^23^, observed positive effects of municipal organic waste compost in arid and semi-arid conditions, supporting the results of the present study. The results clearly indicate the positive impact of organic amendments, particularly vermicompost, on macronutrient content and uptake in onion plants (Table 4) grown in calcareous soils. Such soils often have low nutrient availability due to high pH and calcium carbonate, which precipitates phosphorus and reduces its availability. The observed increases in N, P, and K contents with rising vermicompost rates can be explained by its slow mineralization, gradually releasing nutrients while enhancing microbial activity in the rhizosphere^20,24,25^). Vermicompost also improves soil physical structure, water holding capacity, and root development, all facilitating better nutrient uptake. Phosphorus availability, a limiting factor in calcareous soils, was notably improved, likely due to humic substances and organic acids in vermicompost chelating calcium and releasing phosphate into the soil solution^26,27^). Potassium uptake also increased, reflecting improved root health and cation exchange capacity. Vermicompost consistently outperformed town refuse, possibly because of its finer particle size, stabilized organic matter, and higher microbial activity, which better synchronize nutrient release with plant demand. Town refuse, while beneficial, contains fewer available nutrients and may have higher compositional variability. Overall, these findings support using vermicompost at 20 ton/fed-1 as a sustainable alternative or supplement to chemical fertilizers to improve onion productivity and quality in calcareous soils. The observed enhancement in micronutrient content and uptake (Table 5) can be attributed to several beneficial effects of organic amendments. Micronutrient availability vermicompost increases the bioavailability of micronutrients by improving soil structure, microbial activity, and chelation of trace elements, reducing their fixation in calcareous soils^28,29^). Both vermicompost and town refuse enhance cation exchange capacity (CEC), locally lower soil pH around the root zone, and increase organic matter content, facilitating micronutrient mobility^30^. Increased Uptake Efficiency enhanced root growth, better root-soil contact, and symbiotic microbial populations improve plant capacity to absorb and translocate Fe, Zn, and Cu, particularly under the chelating influence of humic and fulvic acids in vermicompost^31^. While town refuse was also effective, it was slightly less efficient than vermicompost, likely due to variability in composition and lower microbial activity. Nevertheless, at 20 ton fed^−1^, town refuse still provided significant improvements over the control. The increase in oil content (Fig. 2) may be attributed to enhanced nutrient availability, particularly potassium and sulfur, which are essential for lipid metabolism and synthesis. Vermicompost, rich in humic substances and microbial activity, likely stimulated metabolic processes related to oil biosynthesis^32^. The rise in TSS reflects improved carbohydrate accumulation, likely due to better root development and enhanced nutrient uptake under organic amendment treatments^33^. Higher protein content indicates improved nitrogen availability and sustained nutrient release from vermicompost and town refuse, facilitating continuous protein synthesis throughout the growing period^20^. Overall, vermicompost at 20 ton fed^−1^ appeared slightly more effective in enhancing biochemical quality, though both amendments significantly improved oil, sugar, and protein contents in onion bulbs.

Potassium (K %) displayed the strongest correlations with all growth traits and quality parameters (oil and TSS), underlining its essential role in osmoregulation, enzyme activation, and sugar transport^34^. Higher K levels likely facilitated increased bulb expansion, carbohydrate accumulation, and oil synthesis. The total leaf area (TLA) was strongly correlated with nutrient content and biochemical traits, indicating that a larger photosynthetic area enhanced assimilate production, contributing to higher protein, oil, and sugar content. The positive association between micronutrients (Fe, Zn, and Cu) and oil/TSS may be due to their roles in enzyme systems, chlorophyll formation, and energy metabolism. Notably, Zn and Cu are co-factors for enzymes involved in carbohydrate and lipid biosynthesis. The strong correlations also support the beneficial impact of vermicompost and town refuse in improving physical soil conditions, microbial activity, and nutrient availability—especially in calcareous soils, which typically suffer from poor structure and nutrient fixation. This correlation analysis reinforces that applying organic amendments not only improves growth performance but also enhances nutritional and quality traits of onion plants, supporting sustainable production strategies.

## Conclusion

The application of vermicompost and town refuse significantly improved the growth performance, nutrient uptake, and quality traits of onion plants cultivated in calcareous soil. Vermicompost, especially at 20 ton fed^−1^, led to the highest increases in chlorophyll content, bulb and leaf biomass, and root length. It also significantly enhanced the uptake of macronutrients (N, P, K) and micronutrients (Fe, Zn, Cu), improving both vegetative and biochemical attributes such as oil content, total soluble sugars, and protein levels. Correlation analysis revealed strong, positive interrelationships between growth traits and nutrient accumulation, particularly potassium and phosphorus, which were tightly linked to bulb development and oil yield. These results underscore the agronomic value of vermicompost as a superior organic input for sustainable onion cultivation in challenging calcareous soils, promoting higher yield and better quality while reducing dependency on chemical fertilizers.

## Data Availability

“All data generated or analysed during this study are included in this published article.”
